# MAXWELL A. POLLACK AWARD FOR CONTRIBUTIONS TO HEALTH AGING PRESENTATION AND LECTURE

**DOI:** 10.1093/geroni/igad104.0398

**Published:** 2023-12-21

**Authors:** Deborah Waldrop

## Abstract

The Maxwell A. Pollack Award for Contributions to Health Aging Lecture will feature an address by the 2022 Pollack Award recipient Nancy Morrow-Howell, MSW, PhD, FGSA, of Washington University in St. Louis. This session will also include the presentation of the 2023 Maxwell A. Pollack Award to recipient Bei Wu, PhD, FGSA, FAGHE of New York University. The Maxwell A. Pollack Award for Contributions to Healthy Aging Award recognizes instances of practice informed by research and analysis, research that has directly improved policy or practice, and distinction in bridging the worlds of research and practice.

